# Functional Prediction of *AT5G35460* Reveals Its Regulatory Role in Reproductive Development and Lipid Remodeling in *Arabidopsis thaliana*

**DOI:** 10.3390/membranes16030088

**Published:** 2026-02-28

**Authors:** Muhammad Asif Shabbir, Mustansar Mubeen, Muhammad Umer, Aqleem Abbas, Amjad Ali, Sarmad Ali Qureshi, Muhammad Junaid Rao, Yasir Iftikhar, Esmael M. Alyami, Ahmed Ezzat Ahmed

**Affiliations:** 1 Department of Plant Pathology, College of Agriculture, University of Sargodha, Sargodha 40100, Pakistan; shabbir.asif696@gmail.com (M.A.S.); mustansar01@yahoo.com (M.M.); 2 School of Breeding and Multiplication (Sanya Institute of Breeding and Multiplication), College of Tropical Agriculture and Forestry, Hainan University, Sanya 572025, China; 3 Department of Plant Pathology, College of Plant Science and Technology, Huazhong Agricultural University, Wuhan 430070, China; 4 Department of Agriculture and Food Technology, Karakoram International University, Gilgit 15100, Pakistan; aqlpath@gmail.com; 5 Department of Plant Protection, Faculty of Agricultural Sciences and Technology, Sivas University of Science and Technology, Sivas 58140, Türkiye; amjadbzu11@gmail.com; 6 Department of Field Crops, Faculty of Agricultural Sciences and Technologies, Sivas University of Science and Technology, Sivas 58140, Türkiye; sarmadq95@gmail.com; 7 State Key Laboratory for Development and Utilization of Forest Food Resources, Zhejiang A&F University, Hangzhou 311300, China; mjr@zafu.edu.cn; 8 Department of Biology, College of Science, King Khalid University, Abha 61413, Saudi Arabia; ealshahi@kku.edu.sa (E.M.A.); aabdelrahman@kku.edu.sa (A.E.A.); 9 Health and Medical Research Centre (HMRC), King Khalid University, Abha 61413, Saudi Arabia; 10 Prince Sultan Bin Abdelaziz for Environmental Research and Natural Resources Sustainability Center, King Khalid University, Abha 61421, Saudi Arabia

**Keywords:** *AT5G35460* gene, glycerophosphocholine acyltransferase, membrane lipid remodeling, pollen development, stress-responsive gene regulation, *Arabidopsis thaliana*

## Abstract

Membrane lipid remodeling plays a pivotal role in regulating plant growth, reproductive development, and adaptive responses to environmental stress. However, several lipid-modifying enzymes remain uncharacterized in *Arabidopsis thaliana*. Here, we provide the first comprehensive in silico functional characterization of the unannotated gene *AT5G35460*, integrating domain architecture, AlphaFold-supported structural validation, and phylogenetic, expression, and regulatory analyses. Domain architecture and conserved DUF2838 signatures, together with transmembrane topology and validation using AlphaFold-predicted structural data, support its identity as a glycerophosphocholine acyltransferase (GPCAT1). Phylogenetic reconstruction showed that GPCAT1 clustered closely with its orthologs of major angiosperms, suggesting deep evolutionary preservation. Expression profiling revealed over a tenfold higher transcript abundance in mature pollen, detected 6–8 times more than during leaf senescence, indicating strong developmental control. Co-expression network analysis revealed links to the lipid metabolism genes (*CDS2*, *LACS8*, and *SBH1*) as well as factors involved in response to stress, indicating that *AT5G35460* may act at the level of phosphatidylcholine remodeling, membrane resistance and stress response. Analysis of the promoter sequences showed AACTAAA, ABRE and G-box elements (pollen-specific, ABA-responsive and stress-inducible motif respectively), suggesting appropriate transcriptional regulation consistent with its expression profile. As a whole, the findings revealed that *AT5G35460* is an unexplored membrane-localized acyltransferase involved in lipid maintenance during reproductive development and environmental responses. This study serves as a basis for subsequent functional characterization and identifies *AT5G35460* as a potential target for modifying pollen viability, senescence kinetics and stress tolerance in plants.

## 1. Introduction

The membranes of plants are not rigid structures but rather dynamic and they change their lipid composition to facilitate growth, reproduction and stress resistance. Phosphatidylcholine (PC) is especially prevalent on membranes and plays a crucial role in maintaining bilayer stability and signaling pathways and being the storage pool for fatty acid remodeling during developmental switching or environmental stress [1,2]. These remodeling reactions are primarily mediated by acyltransferases such as lysophosphatidylcholine acyltransferases (LPCATs) and glycerol-3-phosphate acyltransferases (GPATs). LPCAT and GPAT enzymes are important regulators of phosphatidylcholine remodeling and reproductive development in *Arabidopsis* [2,3,4]. However, glycerophosphocholine acyltransferase (GPCAT) represents an alternative acyl-editing pathway that remains genetically underexplored in plants. These examples highlight how fine-tuned lipid modification is critical for reproductive success. GPCAT reacylates glycerophosphocholine (GPC) to produce lyso-PC and PC instead of the classical CDP-choline pathway. This activity has been demonstrated in yeast, *Arabidopsis* microsomes and developing oil seeds, where it contributes to membrane lipid remodeling and energy efficiency [5]. Despite its importance, the genetic basis of GPCAT activity in plants remains largely unexplored. The *Arabidopsis* gene *AT5G35460* has attracted interest because public transcriptome datasets show its strong expression in pollen and senescing leaves and under certain stress conditions [6]. Still, its molecular function, evolutionary relationships and regulatory features are unknown. We hypothesize that *AT5G35460* encodes a glycerophosphocholine acyltransferase (GPCAT) based on conserved DUF2838 domain architecture and its pollen- and senescence-associated expression patterns. No study has provided a systematic analysis combining structural predictions, phylogenetics, gene expression profiling, promoter motif discovery, and co-expression networks to evaluate its biological role. Understanding such uncharacterized genes is more than an academic exercise. Lipid remodeling pathways are emerging as key targets for improving plant stress resilience and reproductive efficiency in crops [7]. Identifying and prioritizing candidate genes through bioinformatics accelerates experimental research and enables functional validation and translational applications.

In this study, the first comprehensive characterization of *AT5G35460* using an integrated bioinformatics approach is being presented. The workflow includes domain architecture analysis, structural validation using AlphaFold-predicted models, subcellular localization prediction, phylogenetic reconstruction, tissue-specific expression profiling, co-expression network analysis and promoter motif identification. Together, these analyses suggest that *AT5G35460* encodes a GPCAT-like enzyme involved in phosphatidylcholine remodeling during pollen maturation, leaf senescence and stress responses. This prediction serves to bridge a significant discrepancy within the context of membrane lipid metabolism in *Arabidopsis*. This study identifies *AT5G35460* as a strong candidate for functional verification and offers not only mechanistic information but also a genetic resource to manipulate plant reproduction development and stress response.

## 2. Materials and Methods

### 2.1. Sequence Retrieval and Gene Annotation

The nucleotide and protein sequences of *AT5G35460* and its two splice variants (*AT5G35460-201* and *AT5G35460-202*) were obtained from The *Arabidopsis* Information Resource (TAIR10; accessed on in December 2024) [8,9]. TAIR was used because it provides the most authoritative and regularly updated *Arabidopsis* genome annotations. Gene structure, chromosomal localization on chromosome 5 and exon–intron organization were verified directly from TAIR to ensure downstream analyses relied on a consistent and accurate reference build.

### 2.2. Conserved Domain Identification and Functional Prediction

Protein domain architecture was analyzed using InterPro 98.0 (accessed in December 2024) [10]. InterPro integrates multiple domain databases to provide consensus annotations. InterPro now integrates Pfam following recent updates. Using this tool minimized false negatives and ensured that even weak or non-canonical motifs were not overlooked. Overlapping predictions from both platforms were cross-validated to confirm motifs consistent with glycerophosphocholine acyltransferase activity.

### 2.3. Subcellular Localization and Structural Modeling

Subcellular localization was predicted using SUBA4 (release 1.2; accessed in December 2024) [11], which was chosen over generic localization predictors such as TargetP because SUBA4 integrates experimental proteomic evidence specific to *Arabidopsis* and increases prediction reliability. Transmembrane regions and topology were examined using TMHMM 2.0 and Phobius 1.01. TMHMM [12] detects hydrophobic helices with high precision while Phobius [13] also identifies N-terminal signal peptides, which allows complementary validation of membrane-associated features. Structural assessment of *AT5G35460* was performed using the AlphaFold-predicted protein structure available for this gene. SWISS-MODEL was used for structural evaluation and visualization rather than de novo model generation [14], as it was preferred for its automated template selection and quantitative scoring (GMQE and QMEAN) to assess stereochemical quality. Templates with the highest sequence identity and coverage were selected, ensuring that only high-confidence models were interpreted.

### 2.4. Phylogenetic and Evolutionary Analysis

Homologous protein sequences from diverse plant species were retrieved from NCBI RefSeq (accessed in December 2024) to assess evolutionary conservation. Multiple sequence alignment was performed using Clustal Omega 1.2.4 [15], which was selected for its accuracy in aligning divergent plant sequences. Phylogenetic trees were constructed in MEGA X 12.0.14 [16] using the maximum likelihood method with the Jones–Taylor–Thornton (JTT) substitution model. Bootstrap analysis with 1000 replicates was performed to assess branch support. MEGA 12 was used because it offers a user-controlled pipeline for evolutionary modeling with strong statistical support.

### 2.5. Expression Profiling and Co-Expression Network Analysis

Tissue-specific and developmental expression patterns of *AT5G35460* were analyzed using ATTED-II v11.0 (accessed in December 2024) [17] and *Arabidopsis* eFP Browser (accessed in December 2024) [18], which provides curated microarray and RNA-seq datasets visualized across organs, developmental stages and experimental treatments. These platforms were chosen because they compile normalized transcriptome datasets and reduce variability between experiments. Co-expression analysis in ATTED-II identified 50 functionally associated genes using mutual rank (MR) scoring [17], providing insight into pathways related to membrane lipid remodeling and stress responses.

### 2.6. Promoter Motif and Regulatory Element Identification

The 2 kb upstream promoter region of *AT5G35460* was retrieved from TAIR and analyzed using PlantCARE (accessed in December 2024) [19] to identify conserved cis-regulatory elements. PlantCARE was selected because it provides curated and plant-specific promoter motifs. The AACTAAA regulatory motif linked to pollen maturation and guard cell activity was highlighted to explain observed tissue-specific expression patterns.

### 2.7. Gene Ontology (GO) Enrichment Analysis

To predict biological processes associated with genes co-expressed with *AT5G35460*, AgriGO v2.0 (accessed in December 2024) [20] was used. AgriGO was selected because its FDR-controlled statistical tests (Fisher’s exact test with FDR < 0.05) are optimized for plant datasets to ensure high-confidence functional annotations. Enriched terms revealed involvement in vesicle-mediated transport, phosphatidylinositol biosynthesis and protein targeting pathways. In addition, KEGG pathway enrichment analysis of the co-expressed gene set was performed using ShinyGO v0.85.1 [21] with *Arabidopsis thaliana* (TAIR10) as the reference background.

## 3. Results

### 3.1. Sequence Features and Domain Organization

**Figure 1:**
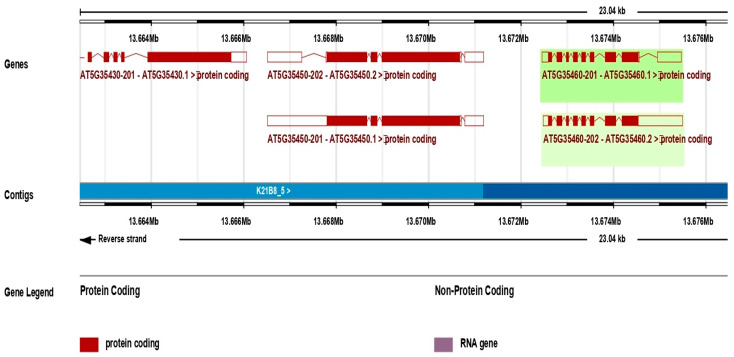
Exon–intron organization of *AT5G35460* (GPCAT1) splice variants in *Arabidopsis thaliana*.

The *AT5G35460* gene of *Arabidopsis thaliana* encodes for glycerophosphocholine acyltransferase 1 (GPCAT1) and it is located on chromosome 5 (13,672,463–13,675,476 bp, forward strand). This protein-coding locus produces two splice variants, *AT5G35460.1* and *AT5G35460.2*, and comprises nine exons separated by eight introns. The coding sequence aligns precisely with the annotated genomic coordinates with exons of ~50–150 bp and introns ranging from several dozen to a few hundred base pairs. Both isoforms retain the DUF2838 domain (IPR021261), which is essential for phospholipid metabolism (Figure 1). Comparison of the two splice variants showed that all four mutants in *AT5G35460.1* and *AT5G35460.2* maintain the entire DUF2838 domain and therefore, they may still have catalytic activity. There were no substantial variations in their predicted domain architecture between the isoforms. Public expression datasets (*Arabidopsis* eFP Browser) show that *AT5G35460.1* is predominant in all tissues, mature pollen and senescing leaves included, whereas *AT5G35460.2* is down-regulated at modest levels and without significant tissue-specific enrichment. This indicates that the main isoform probably mediates functional response. The CDS begins with an ATG start codon and ends with a TGA stop codon and introns display canonical GT–AG splice sites. Alternative splicing likely contributes to functional variation across tissues. Expression data show activity in mature pollen, leaves, cotyledons, roots and floral organs that is consistent with a role in phosphatidylcholine biosynthesis. The defined exon–intron structure provides a basis for investigating transcriptional regulation and functional diversity of its isoforms.

### 3.2. Conserved Domain Results and Functional Insights 

InterPro analysis of the *AT5G35460* protein (381 amino acids) identified a single conserved domain, PF10998 (DUF2838), spanning residues 67–176 with a highly significant E-value of 5.5 × 10^−35^. Although it has been reported as a domain of unknown function, cross-referencing with Pfam and PANTHER linked it to glycerophosphocholine acyltransferase 1 activity. The predicted function was further supported by association with multiple MetaCyc pathways, including PWY-3602, PWY-361, PWY-4801, PWY-4922 and PWY-5048, and all pathways are related to glycerophospholipid metabolism. These findings indicate that *AT5G35460* encodes a putative membrane-associated acyltransferase involved in phospholipid remodeling, consistent with its conserved domain architecture.

### 3.3. Subcellular Localization and Structural Results 

**Figure 2:**
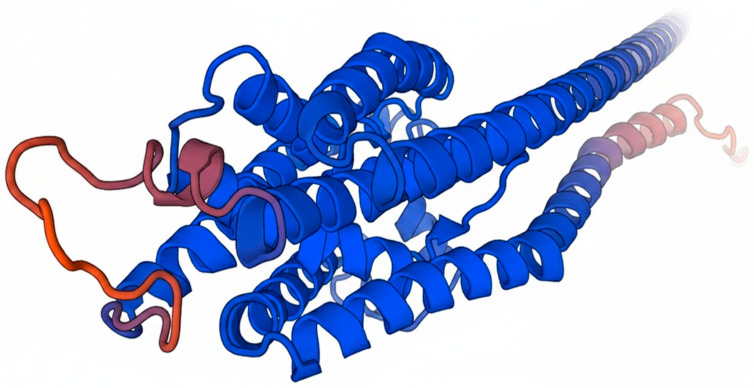
SWISS-MODEL 3D homology model of *AT5G35460* highlighting catalytic domain orientation and membrane-anchoring helices. Colors represent the confidence level of the predicted structure, with blue indicating high confidence and warmer colors (yellow to red) indicating lower confidence regions.

Subcellular topology prediction using TMHMM and Phobius identified multiple hydrophobic segments, indicating that *AT5G35460* is a multi-pass integral membrane protein with alternating cytoplasmic and non-cytoplasmic loops (Figure 2). PANTHER classification placed the sequence within family PTHR31201, and no additional conserved motifs or repeats were detected (Figure 2).

**Figure 3:**
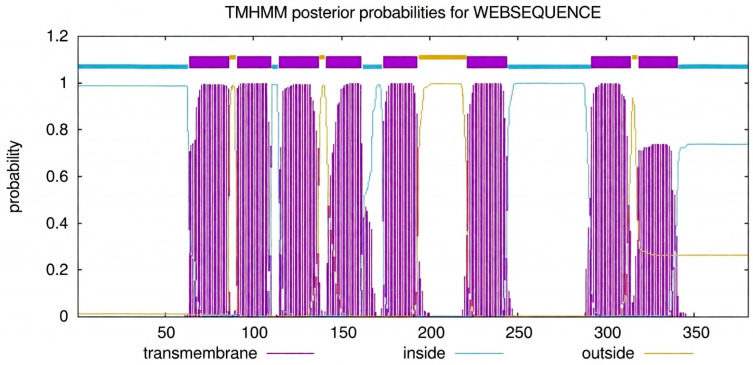
TMHMM posterior probability plot showing predicted transmembrane helices and orientation of *AT5G35460* within the membrane.

Functional annotation linked the protein to the phosphatidylcholine biosynthetic process (GO:0006656) and multiple lipid metabolism pathways in MetaCyc. The AlphaFold-predicted structure of *AT5G35460* was retrieved and assessed using SWISS-MODEL quality metrics (GMQE 0.86), confirming agreement with the predicted transmembrane architecture (Figure 3). Two additional low-identity, partial-coverage models (GMQE 0.04) were generated from unrelated templates but were considered unreliable. The combined topology and structural evidence support the classification of *AT5G35460* as a membrane-bound glycerophosphocholine acyltransferase likely to function in phospholipid remodeling. 

### 3.4. Evolutionary Conservation and Phylogenetic Analysis

**Figure 4:**
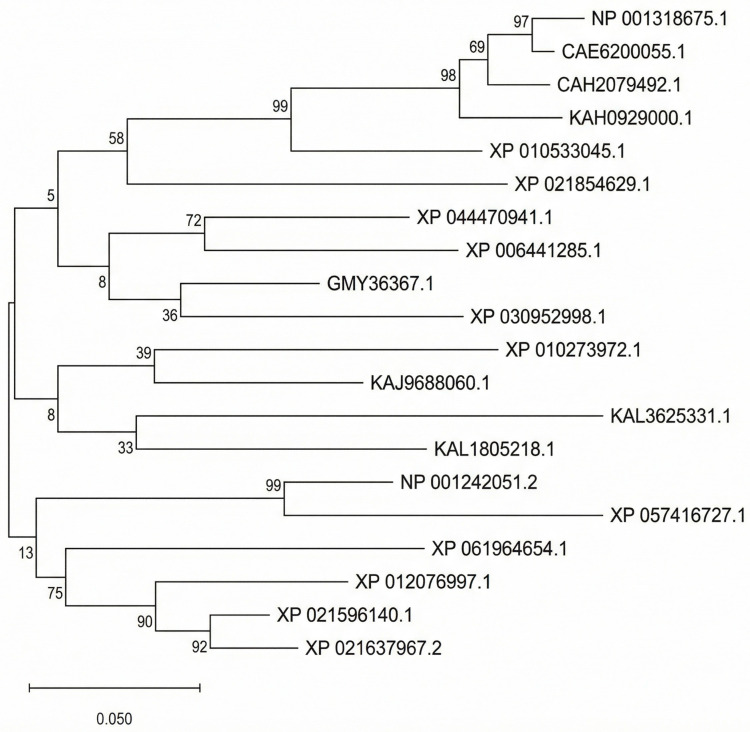
Maximum likelihood phylogenetic tree of *AT5G35460* and GPCAT1 homologs from diverse plant species constructed using the JTT substitution model. Bootstrap support values (1000 replicates) are shown at the nodes.

Homologous protein sequences of *AT5G35460* were found in different plant species: *Brassica napus*, *Oryza sativa*, and *Zea mays* (Figure 4). The aligned regions are highly conserved, notably in the predicted catalytic domain as visualized by Clustal Omega multiple sequence alignment. Phylogenetic reconstruction based on maximum likelihood in MEGA12X placed *AT5G35460* into a clearly defined clade of plant glycerophosphocholine acyltransferase1 (GPCAT1) orthologs. The phylogenetic relationships were supported by moderate to high bootstrap values exceeding 90% across key nodes, which indicated the robust phylogenetic resolution. The close clustering of *AT5G35460* with orthologs from both monocot and dicot species suggests that its function has been maintained throughout plant evolution. Phylogenetic conservation also suggests that *AT5G35460* may encode a rate-limiting enzyme in glycerophospholipid metabolism, the evolutionary pressure retaining its structural and functional identity.

### 3.5. Expression Dynamics and Co-Expression Relationships

Tissue-specific and developmental patterns of expression of *AT5G35460* were investigated in ATTED-II v11. 0; *Arabidopsis* eFP Browser and Genevestigator (all accessed in December 2024). These databases offer standardized transcriptome datasets and well curated visualizations, which eliminate the heterogeneity across experiments. The highest expression of *AT5G35460* was identified in mature pollen and senescing leaves from *Arabidopsis thaliana* eFP Browser [18]. Transcript levels were maximal at late pollen development and up-regulated during leaf senescence, both stages with a need for active membrane lipid remodeling. This pattern supports a potential role in maintaining membrane stability and facilitating phosphatidylcholine turnover during developmental transitions. Co-expression analysis using ATTED-II identified 50 genes with strong expression correlation (Mutual Rank < 20). These include genes such as *CDS2* (cytidinediphosphate diacylglycerol synthase 2), *SBH1* (sphingoid base hydroxylase 1), *BI1* (BAX inhibitor 1), *LACS8* (long-chain acyl-CoA synthetase 8) and *LPPγ* (phosphatidic acid phosphatase family protein), which are all involved in lipid biosynthesis, vesicle trafficking and membrane remodeling. Several co-expressed genes (Table 1) also participate in abiotic and biotic stress responses, suggesting that *AT5G35460* may be associated with networks involved in lipid metabolism and environmental adaptation (Table S1: Co-expressed genes associated with *AT5G35460* (MR < 20).

### 3.6. Promoter Motif Analysis and Regulatory Insights 

**Figure 5:**
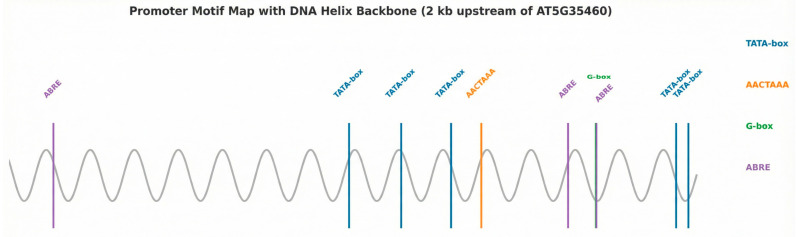
Cis-regulatory elements in the 2 kb promoter region of *AT5G35460*, with the pollen-related AACTAAA motif highlighted.

Scanning of the 2 kb upstream promoter region of *AT5G35460* (Chr5: 13,671,863–13,676,103; TAIR10) revealed several conserved cis-acting elements. An AACTAAA motif previously associated with pollen-related transcriptional regulation was identified at 490 bp relative to the transcription start site (TSS). Although present as a single occurrence, its co-existence with additional regulatory elements such as ABRE and G-box motifs suggests potential combinatorial transcriptional control. Multiple TATA-box motifs (TATAAA) were detected, including two core promoter-proximal elements at −47 bp and −19 bp likely corresponding to basal transcription machinery binding sites. In addition, a G-box motif (CACGTG) was located at 230 bp and ABRE core motifs (ACGTG) were observed at −293 bp and −229 bp, suggesting potential regulation by bZIP/bHLH transcription factors and abscisic acid or stress signaling pathways. These motif signatures support the hypothesis that *AT5G35460* expression is influenced by both tissue-specific transcriptional programs and stress-responsive regulatory networks (Figure 5).

### 3.7. Gene Ontology (GO) Enrichment Results

Gene Ontology (GO) enrichment of genes co-expressed with *AT5G35460* revealed significant associations with vesicle-mediated transport (GO:0016192), protein targeting to membranes (GO:0006612), and phosphatidylinositol biosynthesis (GO:0006661). These enriched categories suggest that *AT5G35460* is associated with a broader co-expression network related to membrane lipid homeostasis and intracellular trafficking. KEGG pathway enrichment analysis for the co-expressed gene set indicated statistically significant overrepresentation of metabolic pathways (FDR = 1.7 × 10^−2^), indicating that *AT5G35460* may be associated with a wider range of metabolic networks. These results are consistent with GO enrichment results and again support that *AT5G35460* is involved in dynamic membrane lipid-related metabolic processes.

## 4. Discussion

This integrative in silico analysis supports the hypothesis that *AT5G35460* (GPCAT1) functions as a glycerophosphocholine acyltransferase, an integral player in phosphatidylcholine (PC) homeostasis and membrane lipid remodeling in *Arabidopsis thaliana*. This interpretation is supported by domain architecture, structural predictions, phylogenetic conservation and expression dynamics. The identification of the DUF2838 domain consistent with glycerophosphocholine acyltransferase activity aligns *AT5G35460* with the biochemical activity of GPCAT as demonstrated in earlier enzymatic studies. GPCAT enzymes use acyl-CoA to acylate glycerophosphocholine (GPC), producing lysophosphatidylcholine (LPC), which is later converted back to PC and contributes to a PC recycling or remodeling pathway [22]. These enzymes exhibit broad acyl specificity and are structurally distinct from other acyltransferases and traits corresponding with our domain analysis. Functional investigations in yeast and plant extracts confirm that GPCAT activity participates in PC biosynthesis and remodeling. For example, labeling studies in yeast confirm that the Gpc1 enzyme (yeast GPCAT) plays a role in PC biosynthesis via GPC reacylation [22]. Moreover, the conservation of this activity in plants underscores its evolutionary importance. Hallström et al. (2022) reported that knockouts of the GPCAT gene in *Arabidopsis* exhibited altered expression of sphingolipid-related genes under cold stress, suggesting GPCAT’s involvement in lipid regulation during low-temperature stress [23]. These observations complement the co-expression analysis that associates *AT5G35460* with lipid metabolism genes such as *LACS8* and *LPPγ* as well as stress-responsive regulators, hinting at a functional role in stress-adaptive membrane remodeling. While the AACTAAA motif was detected as a single instance, its presence alongside ABA-responsive and stress-related elements indicates a likely role in coordinated regulatory activity rather than as an independent determinant of pollen specificity. The Gpc1 enzyme is essential in a post-synthetic PC deacylation/reacylation pathway (PC-DRP) in *Saccharomyces cerevisiae*, altering the saturation profile of PC species. Loss of Gpc1 induces shifts in PC composition and compromises stationary-phase viability [24], while in *Candida albicans*, Gpc1 knockout affects PC levels, hyphal growth and long-term viability, emphasizing its functional importance across eukaryotes. These findings support the notion that GPCAT family members, including *AT5G35460*, may be critical for membrane remodeling, developmental transitions and viability. The presence of two splice variants that both retain the catalytic domain further supports functional conservation of *AT5G35460* activity. High levels of *AT5G35460* transcripts are observed in mature pollen and senescing leaves, tissues where rapid membrane rearrangement and turnover occurs. This suggests that GPCAT1-generated PC remodeling might fulfill the specific demand for increased membrane dynamics during reproductive development and programmed senescence, both times when acyl-editing and control of membrane fluidity are most critical. Indeed, PC acyl-editing enables the property-maintaining capability under changing physiological conditions of membrane systems, and as an ulterior model, *AT5G35460* catalyzes GPC-E2 formation to control pool integrity (acyl chain variety) and the flexible response to developmental cues and environmental stress [25].

## 5. Conclusions

Bioinformatics-based observations, supported by the scientific literature, expression specificity and evolutionary conservation as well as reported functions of GPCAT enzymes, support the classification of *AT5G35460* as an orphan GPCAT1 homolog and its identification as an active GPCAT1 homolog in *Arabidopsis thaliana*. It is located at a critical branch point for PC acyl-editing, membrane homeostasis and developmental viability. Comparing computations to the available experimental literature serves to illustrate both the validity and importance of functional validation of computational predictions.

## Figures and Tables

**Table 1:** Top 50 co-expressed genes with *AT5G35460* identified by ATTED-II (Mutual Rank < 20) related to lipid remodeling and stress responses.

Rank	Gene Symbol	Locus ID	Annotation	MR	Functional Category
1	*AT4G13010*	At4g13010	Zinc-binding dehydrogenase	6.6	Redox metabolism
2	*SBH1*	At1g69640	Sphingoid base hydroxylase	6.2	Sphingolipid metabolism
3	*LACS8*	At2g04350	Acyl-CoA synthetase	5.7	Lipid metabolism
4	*BI1*	At5g47120	BAX inhibitor	5.6	Stress response
5	*AT5G64170*	At5g64170	DSP-like protein	5.5	Unknown/membrane-associated
6	*BTI2*	At4g11220	VirB2 interacting protein	5.5	Vesicle trafficking
7	*REF*	At1g67360	Rubber elongation factor	5.5	Lipid storage/metabolism
8	*CRL1*	At2g33590	Rossmann-fold protein	5.3	Enzyme activity
9	*MIPS2*	At2g22240	Myo-inositol synthase	5.3	Membrane precursor synthesis
10	*TRX-like1*	At1g07700	Thioredoxin protein	5.2	Redox regulation
11	*AT1G22750*	At1g22750	Unknown protein	5.2	Unknown
12	*NAC069*	At4g01550	NAC transcription factor	5.0	Developmental regulation
13	*AGP21-like*	At5g11680	Arabinogalactan protein	4.9	Cell wall/reproduction
14	*BTI1*	At4g23630	VirB2 interacting protein	4.8	Vesicle trafficking
15	*AT4G10430*	At4g10430	TMPIT-like protein	4.8	Unknown
16	*ARD4*	At5g43850	Cupin protein	4.6	Stress metabolism
17	*AT2G38740*	At2g38740	HAD hydrolase	4.6	Metabolism
18	*AT5G67140*	At5g67140	F-box protein	4.5	Protein turnover
19	*ECT8*	At1g79270	RNA-binding protein	4.5	Gene regulation
20	*HISRS*	At3g10250	Histidine tRNA ligase	4.5	Protein synthesis
21	*SBT-like*	At2g33585	Subtilisin protease	4.4	Protein processing
22	*AT4G11570*	At4g11570	HAD hydrolase	4.3	Metabolism
23	*AT5G57610*	At5g57610	Kinase domain protein	4.3	Signal transduction
24	*RING-like*	At3g61180	RING protein	4.3	Ubiquitination
25	*GR1*	At3g24170	Glutathione reductase	4.3	Stress tolerance
26	*AT1G73480*	At1g73480	Hydrolase	4.2	Metabolism
27	*AT5G46170*	At5g46170	F-box protein	4.2	Protein turnover
28	*AT5G65480*	At5g65480	Unknown protein	4.2	Unknown
29	*SK1*	At2g21940	Shikimate kinase	4.2	Secondary metabolism
30	*CLPB3*	At5g15450	Caseinolytic protease	4.1	Protein folding
31	*LPPγ*	At5g03080	Lipid phosphatase	4.1	Lipid metabolism
32	*AT5G15910*	At5g15910	Rossmann protein	4.0	Metabolism
33	*ABA1*	At5g67030	ABA biosynthesis enzyme	4.0	Stress hormone pathway
34	*AT5G51740*	At5g51740	Peptidase	4.0	Protein turnover
35	*AT3G17800*	At3g17800	tRNA ligase-like	4.0	Translation
36	*RER1B*	At2g18240	ER retention protein	4.0	Protein trafficking
37	*AT1G13360*	At1g13360	Unknown protein	3.9	Unknown
38	*CCR1*	At1g15950	Cinnamoyl-CoA reductase	3.9	Phenylpropanoid pathway
39	*AT1G66900*	At1g66900	Hydrolase	3.9	Metabolism
40	*AT2G23780*	At2g23780	RING protein	3.9	Ubiquitination
41	*PP2-A15*	At3g53000	Phloem protein	3.8	Transport
42	*DJA-like*	At1g21660	Chaperone protein	3.8	Protein folding
43	*AT3G07090*	At3g07090	Thiol peptidase	3.8	Protein processing
44	*RKD-like*	At1g20640	Transcription regulator	3.8	Development
45	*APRL7*	At5g18120	Sulfur metabolism	3.8	Stress metabolism
46	*AT1G02270*	At1g02270	Endonuclease	3.8	DNA repair
47	*AT4G28480*	At4g28480	Heat shock protein	3.8	Stress response
48	*CDS2*	At4g22340	CDP-DAG synthase	3.7	Lipid biosynthesis
49	*TPR4*	At1g04530	TPR protein	3.7	Protein interaction
50	*GRP-like*	At4g22740	Glycine-rich protein	3.7	Cell wall/stress

## Data Availability

The raw data supporting the conclusions of this article will be made available by the authors, without undue reservation.

## Supplementary Materials

The following supporting information can be downloaded at: https://www.mdpi.com/article/10.3390/membranes16030088/s1, Table S1: Co-expressed genes associated with *AT5G35460* (MR < 20).
