# Supplementary material for: Functional Prediction of AT5G35460 Reveals Its Regulatory Role in Reproductive Development and Lipid Remodeling in Arabidopsis thaliana
Source: Membranes (Basel). 2026 Feb 28;16(3):88. doi: 10.3390/membranes16030088 (PMC13027849; doi:10.3390/membranes16030088)
Supplement: Supplementary file 1 [file membranes-16-00088-s001.zip › membranes-4150646-supplementary.pdf]

Table S1. Co-expressed genes associated with AT5G35460 (MR < 20)

AT5G35460	AT5G66250	AT1G48790
AT2G45980	AT3G30390	AT1G49300
AT4G31450	AT3G54620	AT1G16180
AT1G79590	AT5G35200	AT3G19420
AT4G28770	AT4G32160	AT2G01600
AT1G32230	AT5G35980	AT4G19860
AT2G01490	AT4G04320	AT1G09920
AT3G48880	AT4G32760	AT2G43160
AT5G49710	AT4G21790	AT5G45410
AT4G19640	AT5G39510	AT4G04800
AT3G05160	AT1G51200	AT3G46060
AT3G02460	AT5G18120	AT4G26060
AT4G22330	AT4G01610	AT3G56950
AT1G67800	AT2G35680	AT5G05930
AT1G22620	AT1G54320	AT5G61530
AT2G38020	AT1G27290	AT1G34190
AT1G10410	AT1G10940	
**Top expressed genes in common between EA/ATTED**		
AT1G09920	AT1G16180	
AT3G30390	AT4G28770	
AT4G31450	AT5G45410	
